# Engaging Adolescents in Using Online Patient Portals

**DOI:** 10.1001/jamanetworkopen.2023.30483

**Published:** 2023-08-23

**Authors:** Bryan A. Sisk, Alison L. Antes, Christine Bereitschaft, Madi Enloe, Sunny Lin, Meghana Srinivas, Fabienne Bourgeois, James M. DuBois

**Affiliations:** 1 Division of Hematology/Oncology, Department of Pediatrics, Washington University School of Medicine, St Louis, Missouri; 2 Bioethics Research Center, Department of Medicine, Washington University School of Medicine, St Louis, Missouri; 3 General Medical Sciences, Department of Medicine, Washington University School of Medicine, St Louis, Missouri; 4 Division of General Pediatrics, Boston Children’s Hospital, Boston, Massachusetts; 5 Department of Pediatrics, Harvard Medical School, Boston, Massachusetts

## Abstract

**Importance:**

Many health care systems offer adolescents access to health information through online patient portals, but few studies have explored how to engage adolescents in using and benefiting from online portals.

**Objective:**

To determine how US children’s hospitals have attempted to encourage adolescent portal use, barriers to engaging adolescents, and ideal future goals for engagement.

**Design, Setting, and Participants:**

This qualitative study performed structured qualitative interviews with informatics administrators from children’s hospitals across the US between February and July 2022. Informatics administrators were employed by US health care systems that managed a children’s hospital with at least 50 dedicated pediatrics beds. Data analysis was performed from November 2022 to January 2023.

**Main Outcomes and Measures:**

This study used thematic analysis of (1) current steps that health care systems had taken to engage adolescents in using their online patient portals and (2) barriers to engaging adolescents and ideal future goals and outcomes of engagement.

**Results:**

Among 58 total interviews with 65 informatics administrators who represented 63 hospitals across 58 health care systems, 6 themes of approaches to engaging adolescents in portal use were identified: (1) promoting and educating adolescents about portal enrollment, (2) establishing workflows to support enrollment, (3) seeking and incorporating feedback, (4) creating a culture or environment supporting engagement, (5) increasing portal utility, and (6) limited efforts. Barriers to engaging adolescents in portal use related to either (1) stakeholder investment, interest, and capabilities or (2) intersecting technical, ethical, and legal factors. Participants identified 4 ideal future efforts to engage adolescents: (1) develop adaptable private means of communication with adolescents, (2) use adolescent-centric user design, (3) enhance promotion and education about portal use, and (4) simplify and adapt workflows to encourage enrollment. Participants described 3 ideal outcomes of this future engagement: (1) provide education about current health, (2) prepare for transition to adulthood, and (3) improve digital health education of adolescents.

**Conclusions and Relevance:**

In this qualitative study of informatics administrators, children’s hospitals across the US were found to have varying degrees of efforts to engage adolescents in using their portals. Most of these efforts focused on supporting adolescent enrollment, but fewer efforts focused on making the portal useful and interesting to adolescents.

## Key Points

**Question:**

What strategies are US children’s hospitals using to engage adolescents in using their online patient portals?

**Findings:**

In this qualitative study, 65 informatics administrators from 58 health care systems described 6 themes associated with institutional approaches to engaging adolescents in portal use. These themes included promotion and education, soliciting feedback from stakeholders, establishing simplified workflows, making the portal more useful for adolescents, and limited or no institutional efforts to engage adolescents.

**Meaning:**

These findings suggest that most efforts of children’s hospitals focus on supporting adolescent enrollment, but fewer efforts focus on making the portal useful and interesting to adolescents.

## Teaser

This qualitative study investigates how US children’s hospitals have attempted to encourage adolescent portal use, barriers to engaging adolescents, and ideal future goals for engagement.

## Introduction

To comply with the 21st Century Cures Act,^1^ many health care systems offer adolescents access to health information through online portals. Portals provide access to results, clinical notes, medication lists, and messaging with clinicians. In adult populations, portal use has been associated with better adherence,^2^ medical understanding,^3,4^ and perceived control over one’s health.^2,3,4,5^ Parents of pediatric patients similarly reported an improved understanding and ability to complete tests and referrals.^6^

Despite these benefits, most scholarship regarding adolescent portals has focused on implications for privacy and confidentiality.^7,8,9,10,11,12,13,14,15,16,17,18^ Of the few studies that explored adolescent engagement with portals, results have been mixed. Kidwell et al^19^ piloted a sickle cell disease–specific portal, finding that portals were acceptable to participants and improved communication. Burkhardt et al^20^ found minimal success using portal messages to encourage COVID-19 vaccination. Allende-Richter et al^21^ piloted an educational intervention to teach adolescents about portals, and adolescents reported more confidence in finding clinical information and increased log-ins.

Portals represent a powerful tool to support adolescent self-management, engagement in health care, and transition to adulthood. However, interventions will only be effective if adolescents use these portals. To our knowledge, no prior work has found how US health care systems are engaging adolescents to use portals. To fill this gap, we interviewed informatics administrators from children’s hospitals and explored current efforts to encourage adolescent portal use, barriers to engaging adolescents, and ideal future goals for engagement.

## Methods

### Participants and Recruitment

This qualitative study followed the Consolidated Criteria for Reporting Qualitative Research (COREQ) reporting guideline.^22^ We performed structured interviews with informatics administrators from US children’s hospitals. Eligibility criteria included: (1) informatics administrators, (2) employed by a US health care system that managed a children’s hospital with at least 50 pediatric beds, and (3) involved in developing or implementing adolescent portal policies. We used the Children’s Hospital Association (CHA) database in January 2022 to identify children’s hospitals. Of 232 hospitals, we excluded specialty or rehabilitation hospitals (n = 37), non-US hospitals (n = 7), and hospitals with less than 50 pediatric beds (n = 9), yielding 179 eligible hospitals. We recruited participants by distributing recruitment materials through 2 informatics email groups and identifying administrators’ contact information through publicly available data. We emailed administrators at every eligible hospital to request interviews. Verbal informed consent was obtained prior to interviews. The Washington University institutional review board approved this study.

### Data Collection

We determined the number of pediatric beds from the CHA database and hospital websites. We developed a structured interview guide that was informed by literature review, formative engagement with informaticians, and feedback from a stakeholder advisory board (eAppendix in Supplement 1). The goal of the interview guide in this study was to examine current adolescent engagement efforts, barriers to engaging adolescents, and ideal future engagement goals. Pertinent to this analysis, we asked, “What steps has your institution taken to engage adolescents in accessing their portal?” In subsequent prompts, we asked about particular clinics or specialties that were more open or less open to engage adolescents, if any standard policies or procedures had been developed, and ideal goals for future engagement. We conducted interviews via telephone or video-conferencing software. B.A.S., a pediatric oncologist and communication researcher with training in qualitative research, conducted interviews between February and July 2022. Interviews were audio recorded and professionally transcribed.

### Data Analysis

We used thematic analysis^23^ of current approaches to engaging adolescents in using online patient portals, as well as barriers to engaging adolescents and ideal future goals to improve engagement. B.A.S. and A.L.A. (1) read transcripts, (2) descriptively coded 5 transcripts to formulate preliminary codes, (3) grouped codes into categories and collapsed categories into representative themes, and (4) refined definitions for themes through 3 cycles of independent coding and consensus meetings. We reached thematic saturation after reviewing 35 transcripts. S.L., M.S., and another researcher independently applied this codebook to all transcripts, reviewed the others’ application of codes, marked disagreements, and resolved disagreements through discussion. All coding was performed using Dedoose qualitative software version 9.0.107 (SocioCultural Research Consultants, LLC). Data analysis was conducted from November 2022 to January 2023.

## Results

### Participant and Health Care System Characteristics

We performed 58 interviews with 65 informatics administrators who represented 63 hospitals, 58 health care systems, 14 379 pediatric beds, and 29 states plus Washington, DC. Interviews ranged from 12 to 43 minutes. The number of dedicated pediatric beds in participating hospitals ranged from 51 to 664 beds (median [IQR], 189 [107-313] pediatric beds). In nonparticipating hospitals, pediatric beds ranged from 50 to 782 (median [IQR], 138 [101-205] pediatric beds) (Table 1).

**Table 1.** Characteristics of Participants and Represented Health Care Systems

Characteristic	No. (%)
Professional role of participant	
Chief medical information officer	34 (52.3)
Clinical informaticist	15 (23.1)
Chief information officer^a^	3 (4.6)
Other^b^	13 (20)
Type of electronic health record	
Epic	41 (70.7)
Cerner	9 (15.6)
Multiple	5 (8.6)
Allscripts	1 (1.7)
Other	2 (3.4)
Pediatric-specific informatics team	
Yes	31 (53.4)
No	27 (46.6)
Pediatric-specific instance of EHR^c^	
Yes	20 (34.5)
No	38 (65.5)
No. of dedicated pediatric hospital beds	
Range	51 to 664
Median (IQR)	189 (107-313)

Abbreviation: EHR, electronic health record.

^a^ Includes 1 participant who identified as director of health information systems.

^b^ Other roles included pediatric service line lead, director of nursing informatics, director of quality, certified analyst, adolescent physician, director of clinical analytics, medical director of informatics, chief medical officer, and clinician champion.

^c^ We defined pediatric-specific instance as a separate build of Epic that was built specifically for use in a pediatric hospital, as opposed to a shared instance where pediatric and adult hospitals share the exact same instance and interface.

### Current Steps to Engage Adolescents in Using Portals

We identified 6 themes of approaches that children’s hospitals used to engage adolescents in portal use. See codebook definitions in Table 2 and excerpts in Table 3. Also, see the Figure for a summary of all themes.

**Table 2.** Codebook Definitions

Parent code	Operational definition
**Current steps to engage adolescents in using portals**	
Promoting and educating adolescents about portal enrollment	Participants described ongoing efforts to promote use of the portal with flyers, videos, and word of mouth. Additionally, they described efforts to educate adolescents about how to enroll in the portal. Many of these efforts relied on clinicians and nursing staff to integrate these activities into their workflow.
Establishing workflows to support enrollment	Participants described efforts to simplify the registration process, ensure support staff are available to help adolescents enroll, and educating staff about enrollment processes. These processes included leveraging clinical needs to incentivize enrollment, such as releasing COVID-19 test results only through portals or requiring portal access to engage in virtual visits.
Seeking and incorporating feedback	Some participants described efforts to seek feedback from adolescents to improve portal access processes. However, other participants described how seeking this feedback was a good idea, but they had not yet done so.
Creating a culture or environment that supports engagement	Some participants described how institutional norms, expectations, and culture prioritized transparency and engagement of adolescents in using their portals.
Increasing portal utility	Participants described the efforts to make the portal useful for adolescents and appealing to their technological and informational preferences.
Limited efforts	Many participants described how their institution had made limited efforts to actively engage adolescents in using the portal.
**Barriers to engaging adolescents in using portals**	
Stakeholder investment, interest, and capabilities	Participants described how successful engagement requires the participation of many stakeholders. Resistance or inability from multiple stakeholders has impeded engagement of adolescents, including adolescents themselves, parents, clinicians, health care systems and/or administration, and politicians.
Intersecting technical, legal, and ethical factors	Participants described how several technical configuration shortcomings in current portals and/or electronic medical records impeded the ability to adequately engage adolescents, such as limited granularity in which data are shared with parents, incorrect contact information, and lack of secure platforms for texting with adolescents. Additionally, these technical limitations led to legal and ethical challenges, especially when parents might access sensitive or protected data.
**Ideal future efforts to engage and outcomes of engagement for adolescents**	
Develop adaptable private means of communication with adolescent	Participants hoped for granular and reliable methods of communicating securely with the adolescent. Ideally, adolescents would be able to determine which information remained private and which information was shared with their parents.
Use adolescent-centric user design for means of communication	Participants described the need to make portals that are appealing to adolescents. If not, then adolescents will not use this technology. Most of these ideals focused on the means of communication, rather than the content.
Enhance promotion and education about portal use	Participants hoped for improved marketing and education to get adolescents interested in using their portals.
Simplify and adapt workflows to encourage enrollment	Participants described the value of incorporating enrollment in the standard clinical workflow to simplify processes and incentivize staff to routinely enroll eligible adolescents. Additionally, some described the goal of a portal-centric culture in which portal use is encouraged and made as easy as possible.
Provide education about current health	Participants hoped that adolescents could use the portal to learn about their current health and become engaged in managing their health.
Prepare for transition to adulthood	In addition to engaging adolescents in managing their current health, participants hoped the portal can be leveraged to help adolescents assume more responsibility and develop autonomy.
Improved digital health education of adolescents	Participants described the ideal of improving adolescents’ ability to navigate the risks of these evolving technologies, perhaps by incorporating digital health and data security into basic education during high school.

**Table 3.** Current Steps and Barriers to Engaging Adolescents in Using Portals

**Theme**	**Example quote**
**Current steps to engage adolescents in using portals**	
Promoting and educating adolescents about portal enrollment	“Adolescents, when they come into their pediatrician’s clinic, they are given a tip sheet and told, ‘If you would like further information, you can either contact [Health Information Management] or speak to your pediatrician.’” (Participant 77)
	“There’s an educational campaign being built that will be a combination of staff education, so front desk or call center staff, when they’re actually scheduling appointments, checking to see if there is an account.” (Participant 52)
Establishing workflows to support enrollment	“We’re working on QR codes in the clinic so that they can help sign up for their MyChart account. We have put a lot of processes in place across the various clinics to help educate people in the availability of MyChart and to get the adolescents signed up, so lots and lots of effort in that direction.” (Participant 26)
	“The pandemic impetus to get people onto our portal was so strong that I think that everyone was trying to do it whenever and wherever possible.” (Participant 119)
Seeking and incorporating feedback	“There is an adolescent advisory group….We’ll bounce our ideas with them, see whether they’re going to be well received or not.” (Participant 164)
	“I don’t think there’s any adolescent voices being represented. I think there’s a lot of parental voices being represented, but I don’t think in our situation, I don’t think that there’s ever been a teen at the table in adolescent practices.” (Participant 144)
Creating culture or environment that supports engagement	“We have really built an entire culture, in our health system, around the use of the patient portal as a primary mode for communication rather than phone, rather than letters.” (Participant 158)
	“I don’t think there’s been focused effort [on adolescents] beyond what’s been done for the population as a whole.” (Participant 78)
Increasing portal utility	“I think that [having the ability to text] will help with sending periodic messages to our teen patients, because we think that’s going to be a successful communication strategy, as opposed to sending emails.” (Participant 184)
	“One thing we were able to do, is we were able to allow e-check-in for the adolescent population. That was a really big way for them to be able to answer the questions on their phone before they come in, so that we could hopefully engage them in those conversations a little bit more.” (Participant 26)
Limited efforts	“We don’t engage well enough yet. I’ll be honest, I think, that’s an area that we need to really develop and we’re gonna work on.” (Participant 140)
	“I don’t think we have as much directed outreach to all of our adolescent patients saying here, look at this video. Do this enrollment process. It’s more if they seek it out.” (Participant 119)
**Barriers to engaging adolescents in using portals**	
Stakeholder investment, interest, and capabilities	“It’s not so much that [clinical staff] don’t want to engage in those conversations with the patients, but I don’t think there’s a lot of time or staff to be able to do that as well.” (Participant 18)
	“Some people are willing to [communicate through the portal], and some are just like, ‘Well, I’ve a got a lot of other priorities in my clinic right now. I’m going to focus on those other than this.’” (Participant 10)
	“To be frankly honest, there is really just not much interest from adolescents, at least in our area, in the patient portal. I think we have probably less than 1 percent of adolescents who actually sign up for a MyChart account and actively use it.” (Participant 103)
Intersecting technical, legal, and ethical factors	“The technology doesn’t support the granularity sometimes. We’ve been able to do that through a lot of work-arounds in our build, but there are many EHR vendors that just frankly don’t allow you to do those things.” (Participant 26)
	“I think in general, we still have a chilling effect on people enthusiastically encouraging teens to enroll in the portal, because people really aren’t sure about the confidentiality. I think our adolescent medicine doctors to get to the back end of your questions I think, some of them actually just tell the kid, I have no idea. I can’t tell you at all that any of this is confidential. You have to assume that your parent might see some stuff, and that’s probably true.” (Participant 153)

Abbreviation: EHR, electronic health record.

**Figure.**
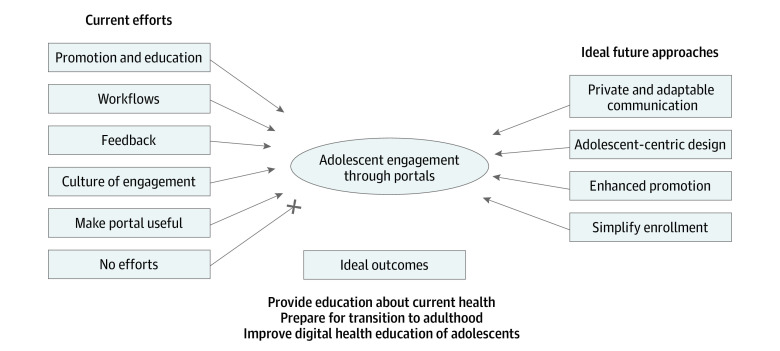
Current Efforts, Future Strategies, and Barriers to Engaging Adolescents Informatics leaders identified 6 themes of current efforts to engage adolescents in using the portal. Most of these efforts focused on improving enrollment, rather than ongoing engagement. Many participants described how their organization had taken little or no action to engage adolescents in using the portal. Participants also described 4 themes of ideal future approaches to engaging adolescents, with 3 themes of ideal outcomes from this engagement.

#### Promoting and Educating Adolescents About Portal Enrollment 

Participants from 25 of 58 health care systems described strategies that focused on promoting portal enrollment, whereas few participants described efforts to improve or incentivize portal use. Many participants described passive approaches, such as distributing flyers, posters, websites, and videos. These approaches relied on clinicians and frontline clinical staff to promote and help patients navigate enrollment. Some centers launched educational campaigns targeting clinical staff to encourage discussions about portal enrollment. With this decentralized approach, clinics varied in how strongly they encouraged adolescent portal use.

#### Establishing Workflows to Support Enrollment

Participants from 16 of 58 health care systems described incorporating enrollment into standard clinical workflows to simplify processes for adolescents and clinicians. For example, some organizations incorporated registration instructions into after visit summaries. Another organization incorporated prompts into clinical note templates to encourage clinicians to discuss portals with adolescents. Other organizations simplified enrollment processes for adolescents to decrease burdens and difficulties of registration, for example, by allowing online enrollment. The COVID-19 pandemic spurred portal enrollment because portals were often required to access telehealth visits and review COVID-19 test results.

#### Seeking and Incorporating Feedback

Participants from 17 of 58 health care systems described the importance of seeking feedback from adolescents to understand what they want or need from portals. Some participants described efforts to obtain this feedback through teen advisory boards and forums. Other participants facilitated research studies to understand the capacity and desire of adolescents to use portals. Furthermore, some hospitals leveraged clinical programs to increase adolescent portal use, such as programs to support transition to adult care. One hospital created a portal satisfaction survey to track adolescents’ impressions and preferences. However, many participants described the absence of adolescent input in developing portal policies and interfaces.

#### Creating a Culture or Environment That Supports Engagement

Participants from 17 of 58 health care systems described institutional campaigns to increase communication through the portal, by which they created expectations that the patient portal is the “primary mode for communication rather than phone, rather than letters” (participant 158). This institutional culture was important because much of the work for enrollment and promotion relied upon frontline clinical staff, which required their buy-in. Other participants described limited or absent institutional efforts to increase adolescent portal use.

#### Increasing Portal Utility

Participants from 9 of 58 health care systems reported leveraging the portal to provide meaningful information to adolescents, such as appointment reminders and vaccine reminders. Certain types of important test results, however, were withheld from the portal due to confidentiality concerns, such as results of pregnancy and sexually transmitted infection tests. Other participants described the benefit of offering mobile portal platforms (rather than only web browser) and developing technology to text with adolescents through the portal. Although some participants described the importance of making portals interesting to patients, few participants described any current approaches to this end. One health care system offered remote check-in through the portal for adolescents to encourage portal use before clinic visits.

#### Limited Efforts

Participants from 19 of 58 health care systems described how their organization had taken limited or no steps to encourage adolescent portal use. Some organizations relied on adolescents to seek out enrollment information. One participant described how adolescent portal usage was low priority, given competing interests: “We really haven’t had enough time to really start to engage adolescents” (participant 56).

### Barriers to Engaging Adolescents in Using Portals

We identified 2 themes of barriers to engaging adolescents in portal use. See excerpts in Table 3.

#### Stakeholder Investment, Interest, and Capabilities

Participants from 40 of 58 health care systems described the challenge of aligning interests of multiple stakeholders to ensure portals were available and useful for adolescents, ranging from administrative staff to nurses and physicians across large organizations. If any stakeholders failed to perform their roles, then portal access became more difficult for adolescents. Time constraints impeded the desire or ability of staff to promote portal use. Furthermore, some clinics were hesitant to adapt or modify their established practice patterns due to inconvenience.

Participants also described challenges with getting adolescents interested in accessing their portals. Institutional decisions to limit portal content further impeded the usefulness of portals for adolescents. These decisions were driven by each institution’s interpretation of state and federal laws.

#### Intersecting Technical, Legal, and Ethical Factors

Participants from 32 of 58 health care systems reported that technical limitations intersecting with ethical and legal expectations created barriers to encouraging adolescent portal access. For example, some organizations did not permit portal access to adolescents based on interpretation of state confidentiality laws. Other participants described barriers posed by lack of configurations to differentially share information between parents and adolescents and concerns about confidentiality, which created a “chilling effect” because clinicians could not guarantee confidentiality to the adolescent (participant 153). Some organizations greatly limited the information sent to the portal to ensure compliance with confidentiality laws, thus limiting the utility of the portal for adolescents. Other organizations protected confidentiality with rigorous identity verification processes. These efforts to prevent parents from using their adolescent’s portal led to onerous enrollment processes that disincentivized enrollment.

### Ideal Future Efforts to Engage and Outcomes of Engagement for Adolescents

Participants described 4 ideal future efforts for how health care systems should engage adolescents. They also described 3 ideal outcomes of portal engagement (Table 4).

**Table 4.** Ideal Future Efforts to Engage and Outcomes of Engagement for Adolescents

**Theme**	**Example quote**
**Ideal efforts to engage adolescents in portal use**	
Develop adaptable private means of communication with adolescent	“In an ideal world, there would be 2 portal accounts. An adolescent account that saw everything and a parent account that was missing all the protected health information that the adolescent should see on their own.” (Participant 93)
	“Make it more autonomous that [adolescents] can manage the access independently. They don’t have to go through us. Do you want them to see meds? Here, have them see meds. Do you want them to see these things, then you can see these things. Here are problem list items. Do you want them to see the problem, these items on your problem list?” (Participant 60)
Use adolescent-centric user design for means of communication	“Then I think engaging in active, trendy ways to get teens to pay attention to things is always a tricky thing, right? [Laughter] Whatever that happens to be today will be something different in 6 months or a year, and so, being able to keep up with that in a way that uses resources highly in the organization.” (Participant 36)
	“Part of the problem is, if your notifications are mostly email, adolescents just don’t live in email. They just don’t like it as much, so as much as it’s more notifications that are text-based notifications—that really the delivery of the information is important for the adolescents to truly engage them. They’re not going to log onto the web-based or desktop version. They really want the mobile versions of all of this.” (Participant 172)
Enhance promotion and education about portal use	“I think it needs to be a concerted marketing campaign that’s probably multimodal. By marketing, I mean within the care team and the clinics, we need to be much more visible. We’ve been talking about this like, ‘Did you know you could do all these things with MyChart?’ Put it out in social media. Have it in the clinic. Have everybody talk to the family about MyChart.” (Participant 155)
	“I personally would have a campaign—you’d have an educational campaign. Part of that educational campaign would be, since we’re dealing with adolescents, maybe a little video. ‘This is what you can do. This is what this information offers you. These are the advantages, and, obviously, this is how you can bring in your proxy if you decide to.’ Yeah, push it. Push it as best and as much as we can.” (Participant 109)
Simplify and adapt workflows to encourage enrollment	“In an ideal world, I would say in an ideal world any time an adolescent is in for a visit there’d be a conversation about, hey, this is a great way to keep in touch with your provider.” (Participant 37)
	“Being able to, I guess, one, make it less confusing to get people signed up and to understand what MyChart is and how it’s the mechanics of getting enrolled.” (Participant 56)
	“I think it really is about helping them, like just doing it, like actually getting them up and running while you’re there, not giving them a handout and say, ‘Hey, download this, and then create a user ID and create password and log-in’. No one does that. No one’s gonna do that.” (Participant 102)
**Ideal outcomes of portal engagement for adolescents**	
Provide education about current health	“I would also love, conversely, to use the portal for education opportunities, specifically on private issues that may be sensitive topics to discuss in clinic, to be able to send it through the portal so that they have that information at their leisure.” (Participant 26)
	“These might be things, it’s like counting steps or other materials to support health and wellness, like managing your exercise or lessons about stretching and nutrition and other things that people might think about—resources for sexual health, resources for nutrition—that would give an adolescent more control over their wellness and other aspects of their nonchronic illness life.” (Participant 158)
Prepare for transition to adulthood	“One of the biggest challenges we have with caring for adolescent patients with chronic disease is that transition to adult care. Some places have good programs for doing it, and other places just wing it. The portal is one of several tools to help people effect that transition. It’s certainly not easy, but it’s an important tool.” (Participant 157)
	“I think it needs to become part of that transitional care conversation. Going from youth to adult transition type of care, starting to do education early on.” (Participant 20)
Improve digital health education of adolescents	“There’s a general need for expansion of social education or digital education that’s part of generally being a citizen. That is part of the public education process, and so, I find that just like we are doing more and more in training adolescents in school on what is a model citizen. What’s appropriate social media, electronic health care record utilization and being a member of that or utilizing that in an appropriate manner makes sense.” (Participant 36)
	“Understanding how to interact with your—understanding what health care management is, how to interact with your provider and what kinds of information you should be caring about, and then understanding what you have rights to and how you can adjust what your parents see or participate in. I would really love to see kind of a comprehensive global education transition plan that was part of their growth and development and part of what we offered as a service of them.” (Participant 139)

#### Ideal Efforts to Engage Adolescents in Portal Use

##### Develop Adaptable Private Means of Communication With Adolescent

Participants from 32 of 58 health care systems called for granular and reliable methods of communicating securely with adolescents. This would permit communication of sensitive results, such as pregnancy or sexually transmitted diseases, to the adolescent through the portal. “To me, it would be my best line of communication almost like an email system where I could send an email to both [parents and adolescents], or I can send an email to 1 of you” (participant 144). Ideally, adolescents would be able to determine which information remained private and which information was shared with parents.

##### Use Adolescent-Centric User Design for Means of Communication

Participants from 27 of 58 health care systems described the need to develop portal interfaces that appeal to adolescents, as well as content or functions that adolescents perceive as useful. If portals are difficult to access or navigate, then adolescents are unlikely to use them. For example, many current portals rely on email to send login information, and many adolescents do not use email. Some participants called for fully mobile platforms to meet adolescents’ preferences. Most responses focused on accessing or enrolling rather than the content of the portal itself. However, 1 participant suggested incorporating interactive games and video education to gain adolescents’ interest.

##### Enhance Promotion and Education About Portal Use

Participants from 15 of 58 health care systems described how enhanced marketing and education could improve the ability and willingness of adolescents to use portals. For organizations that have made limited efforts to engage adolescents, any promotional efforts were viewed as an improvement of current state. Others called for education about portal functions and promotion through social media to reach adolescents. While many organizations had general educational or promotional materials, few had adolescent-specific materials.

##### Simplify and Adapt Workflows to Encourage Enrollment

Participants from 10 of 58 health care systems described the value of incorporating enrollment into standard clinical workflows to incentivize staff to enroll adolescents. Some described the goal of a portal-centric culture in which portal use is encouraged and made easy. Simplification of the logistics of enrollment could further encourage portal access. Another participant emphasized the need to finish enrollment while the patient is in clinic, rather than providing a handout and hoping they would enroll later.

#### Ideal Outcomes of Portal Engagement for Adolescents

##### Provide Education About Current Health

Participants from 5 of 58 health care systems believed that improved engagement strategies could help adolescents to learn and become engaged in managing their current health. Portals could provide an opportunity to learn about sensitive topics that adolescents feel uncomfortable discussing in person. Others described how portals could help adolescents to develop an agenda for clinic visits and get the information and understanding they need from clinicians. Incorporating data from wearables into portals could give adolescents insights into how daily routines affect their health.

##### Prepare for Transition to Adulthood

Participants from 13 of 58 health care systems also hoped that portals could help adolescents assume more responsibility and develop autonomy as they transition to adulthood. Participants described how transitioning adolescents to ownership of care is a persistent challenge, especially for adolescents with chronic illness. Portals could be incorporated into larger initiatives to support adolescents’ readiness for self-management. Portals could become tools for teaching adolescents lifelong skills about managing their health.

##### Improve Digital Health Education of Adolescents

Participants from 5 of 58 health care systems described the importance of using portals as part of an initiative to teach adolescents about digital health and data security. Participants described this education as overlapping with health care transition programs. Others described how portals and digital health care could be 1 component of basic educational curricula during high school. In this way, engaging in portal use would be part of a larger agenda to improve data citizenship. This type of education was viewed as important because rapidly evolving technologies create new risks to confidentiality, safety, and well-being.

## Discussion

Online patient portals represent widely available tools that can support communication, education, and self-management for adolescents. To achieve these goals, however, health care systems must strive to engage adolescents in using portals over time. Informatics administrators in this study described approaches to engaging adolescents in portal use, barriers to this engagement, and ideal future strategies and goals. However, few participants described efforts to make the portal more useful or appealing to adolescents (Figure). These strategies included releasing important test results through the portal (eg, COVID-19 test results) and leveraging the expansion of telehealth during the pandemic to increase enrollments. Additionally, 1 health care system developed a secure texting technology to communicate in the way that many adolescents prefer. While these approaches hold promise, prior efforts to leverage the online portal to increase engagement in care have failed. For example, studies have attempted to leverage the portal to increase vaccination rates,^20^ contraceptive use,^24^ and address high-risk behaviors^25^ with minimal success. Although these proposed strategies are good starting points, this field must develop novel and innovative solutions to increase adolescent engagement.

Registering for portal access is likely insufficient to ensure that adolescents benefit from these technologies. Health care systems must find ways to help adolescents perceive value in accessing their portals. Participants described the importance of seeking and incorporating feedback from adolescents. By engaging adolescents in modifying existing tools, vendors and health care systems could better meet the needs and desires of adolescents. Furthermore, organizations might strive to better leverage existing infrastructure within current electronic health record systems to maximize benefits for adolescents and dissemination of interventions. For example, 1 group previously developed and piloted a disease-specific conditions page within an electronic medical record (Epic MyChart) for adolescents with sickle cell disease, finding that the portal improved perceptions of communication.^19^ This medical record also includes an adaptable add-on application called Care Companion, which can incorporate push notifications, medication reminders, video- and text-based educational materials, and symptom surveys. By engaging adolescents in modifying existing tools or creating new applications, health care systems might increase the utility and appeal of these portals to adolescents.

One major goal described by participants was supporting transitions to adulthood and self-management. This transition is influenced by factors at individual, family, community, and health care system levels.^26,27^ Facilitating communication between adolescents, parents, and clinicians is central to supporting transitions.^28,29,30^ A systematic review found that “across diagnoses, parents who were available to monitor, be a resource, collaborate with their adolescent, and engage in ongoing dialogue were key in the successful transition to autonomous illness management.”^30^ Thus, leveraging portals to support adolescent self-management will require collaborative communication and care management between the adolescent and parents. However, many health care systems provide parents with limited or no proxy portal access due to confidentiality concerns, interpretation of state privacy laws, and lack of technical configuration to differentially share information between parent and adolescent portals.^31^ To realize the potential benefits of portal use for adolescents as they transition to adulthood, organizations must develop strategies to include parents in care when appropriate while protecting adolescent privacy.

A large proportion of participants reported that their institutions had made little or no efforts to engage adolescents in portal use. Informatics administrators have multiple responsibilities, and many organizations were still adapting to the Cures Act mandate at the time of these interviews. Furthermore, many administrators were understandably preoccupied with determining portal access and privacy policies, which could have limited attention to developing robust strategies or efforts to engage adolescents. Online portals are a permanent fixture in adolescent health care, and they offer great potential to improve communication, transitions to self-management, and health outcomes for adolescents. Achieving these goals, however, will require prioritization, intentional planning, and incorporation of adolescents in development of these strategies and interfaces. Furthermore, improving proxy access for adolescents’ caregivers could provide benefits that extrapolate to other populations, such as geriatrics.

### Limitations

This study had limitations. We limited enrollment to hospitals with at least 50 dedicated pediatric beds, which could underrepresent the approaches to adolescent engagement of hospitals with smaller pediatric presence. Participating hospitals were slightly larger than nonparticipating hospitals. We did not include health systems that only managed outpatient care, which could have different experiences than systems including pediatric hospitals. We did not ascertain the clinical roles or training of informatics administrators, which could have influenced their responses or insights. Our results could be biased toward larger pediatric hospitals. Furthermore, participants could have moderated their responses during interviews due to social desirability bias.

## Conclusions

In this qualitative study of informatics administrators, children’s hospitals across the US demonstrated varying degrees of efforts to engage adolescents in using their portals. Most of these efforts focused on supporting adolescent enrollment, but fewer efforts focused on making the portal useful and interesting to adolescents. Participants described ideal future goals that included simplifying enrollment processes and using adolescent-centric user designs to support adolescent education and transitions to self-management. Successful strategies will likely need to incorporate a role for parents in helping adolescents to develop their autonomy.

## Notes

Supplement 1. **eAppendix.** Informatics Experts Interview Guide

Supplement 2.
Data Sharing Statement
