# Supplementary material for: Engaging Adolescents in Using Online Patient Portals
Source: JAMA Netw Open. 2023 Aug 23;6(8):e2330483. doi: 10.1001/jamanetworkopen.2023.30483 (PMC10448298; doi:10.1001/jamanetworkopen.2023.30483)
Supplement: Supplement 2. — Data Sharing Statement [file jamanetwopen-e2330483-s002.pdf]

Data Sharing Statement

Sisk. Engaging Adolescents in Using Online Patient Portals. *JAMA Netw Open*. Published August 23, 2023. doi:10.1001/jamanetworkopen.2023.30483

Data
Data available: Yes
Data types: Deidentified participant data, Data dictionary
How to access data: Request for data must be sent to Bryan Sisk, MD at siskb@wustl.edu.
When available: With publication

Supporting Documents
Document types: Informed consent form
How to access documents: Request for data must be sent to Bryan Sisk, MD at siskb@wustl.edu.
When available: With publication

Additional Information
Who can access the data: Researchers whose proposed use of data has been approved
Types of analyses: For any purpose that is scientifically valid
Mechanisms of data availability: After signed data access agreement
Any additional restrictions: Must have institutional IRB approval and data usage agreement prior to receiving data.
